# The value of endoscopically-placed metal clips for transcatheter arterial embolization in the treatment of recurrent acute non-variceal upper gastrointestinal bleeding

**DOI:** 10.1186/s12876-023-03034-5

**Published:** 2023-11-16

**Authors:** Yinong Zhu, Wenjuan Yang, Yuyan Zhang, Liansong Ye, Bing Hu

**Affiliations:** Department of Gastroenterology, West China Hospital, Sichuan University, No. 37, GuoXue Xiang, Chengdu, 610041 People’s Republic of China

**Keywords:** Metal clip, Acute nonvariceal upper gastrointestinal bleeding, Transcatheter arterial embolization, Angiography

## Abstract

**Objective:**

Acute nonvariceal upper gastrointestinal bleeding (ANVUGIB) is a common clinical emergency. Transcatheter arterial embolization (TAE) is usually used to locate the bleeding site and provide interventional embolization. During TAE, there is a low positive rate of angiography, and localization of the culprit vessel is difficult. The purpose of this study was to demonstrate the role of preplaced metal clips in TAE for ANVUGIB patients.

**Materials and methods:**

Patients with ANVUGIB in whom bleeding sites were identified endoscopically and treated with TAE from January 1^st^, 2005 to July 1^st^, 2021 were retrospectively included. According to the presence or absence of preplaced metal clips, they were divided into two groups. The main outcome measurements included the clinical success rate and rebleeding rate. Secondary outcome measurements included the mortality rate and the need for surgery. Predictors of the clinical success rate were assessed with univariate analysis and multivariate analysis.

**Results:**

A total of 102 patients were included in this study, and all of them had undergone arterial embolization. There were 73 cases in the group with metal clips and 29 cases in the group without metal clips with consistent baseline information. The group with metal clips had a higher clinical success rate (82.2% vs. 45.0%, *P* < 0.001), lower rebleeding rate (8.2% vs 27.6%, *P* = 0.039) and additional surgery rate (11.0% vs 20.7%, *P* < 0.001) than the group without metal clips. In univariate analysis, ROCKALL score and preplaced metal clip marking were shown to affect clinical success rate. In multivariate analysis, metal clip marking was found to facilitate clinical success (OR = 3.750, 95CI = 1.456–9.659, *P* = 0.004).

**Conclusion:**

In ANVUGIB patients, preplaced metal clips could improve the clinical success rate of TAE and reduce the mortality rate and the risk of rebleeding.

## Introduction

Acute nonvariceal upper gastrointestinal bleeding refers to bleeding in the digestive tract above the ligament of Treitz and is caused by nonvariceal diseases. It also includes bleeding in the pancreatic duct or bile duct and diseases near the anastomosis after gastrojejunostomy [1]. ANVUGIB is a common clinical emergency; its annual incidence rate is 19.4 ~ 57.0 per 100,000, and the fatality rate can reach 8.6%. Patients with ANVUGIB should undergo endoscopy or endoscopic treatment within 24 h after bleeding if conditions permit. However, the rebleeding rate after endoscopic treatment is still high, reaching 15–20% [2]. For refractory ANVUGIB with failed endoscopic hemostasis, transcatheter arterial embolization (TAE) is safe and feasible, with technical and clinical success rates of 69%-100% and 63%-67%, respectively. Due to its low invasiveness, short operation time, quick effects, and good patient compliance, TAE has been the common therapy for refractory ANVUGIB in many medical institutions, especially in high-risk patients, and has become the first-line alternative to surgical procedures [2, 3].

**Fig. 1:**
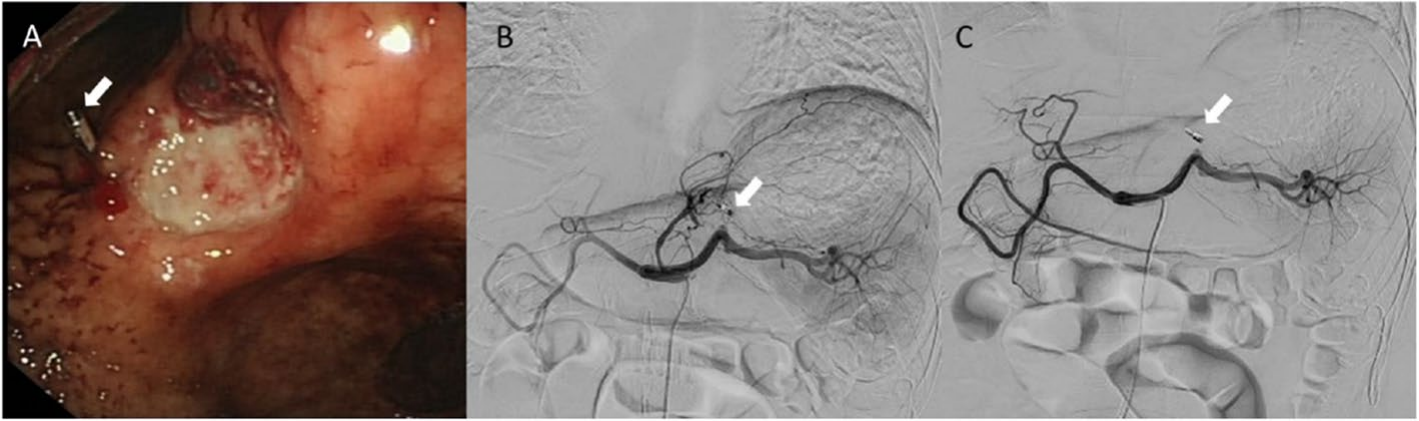
TAE after endoscopic metal clip positioning: **A** Endoscopically, a 1.2 × 1.0cm ulcer in the middle of the gastric horn with a suspected vascular stump, marked with a metal clip (white arrow) at its margin. **B** No spillage of contrast agent during angiography, metal clip seen (white arrow). **C** Embolization of left gastric artery guided by metal clip (white arrow). No postoperative visualization of the target vessel

Before embolization, angiography is required to find the bleeding site where the contrast agent extravasates. However, it may not be possible to see the contrast agent spill if bleeding was due to venous hemorrhage or if bleeding has stopped. In this circumstance, empirical embolization of the left gastric artery or gastroduodenal artery is usually undertaken. However, the blood supply of the pylorus and duodenum is complicated, and the collateral circulation between the celiac trunk and the superior mesenteric artery branches varies greatly. Empirical embolization is not usually effective with a rebleeding rate reaches up to 16% in patients with negative angiographic results [4]. Since most ANVUGIB patients undergo endoscopy before TAE, it has been proposed that the metal clip can be placed at the site or edge of bleeding points during endoscopy to guide embolization during TAE based on the radiopacity of the metal clip. (Fig. 1).

Currently, there are several case reports about the role of metal clips in the TAE of ANVUGIB patients marked with metal clips [5, 2, 6]. No controlled studies on the value of metal clips in TAE have been reported (Table 1). These studies have preliminarily suggested that marking the bleeding site with a metal clip can help locate the bleeding point and guide embolization in patients with ANVUGIB who have failed endoscopic treatment. Metal clip marking could also reduce the occurrence of complications (organ ischemia, necrosis, dislocation of coils, etc.).

**Table 1:** Research status of metal clips in TAE operations in patients with ANVUGIB

Author	Study design	Average age	Positive angiography rate	Technical success rate	Clinical success rate	Rebleeding rate	Reintervention rate	complication
Eriksson et al. [5] *N* = 10	prospective	74.5	40%	90%	80%	/	20%	0
Song et al. [2] *N* = 16	retrospective	59.4	43.8%	100%	87.5%	12.5%	6.3%	0
Wang et al. [6] *N* = 18	retrospective	63.6	55.6%	100%	94.4%	5.6%	11.1%	0

This retrospective study aimed to explore the value of endoscopic metal clip marking at the site of bleeding in TAE for patients with ANVUGIB. Moreover, univariate analysis and multivariate analysis were conducted to evaluate influencing factors of the clinical success rate of TAE.

## Materials and methods

### Study design

We performed a retrospective review of patients with ANVUGIB in whom bleeding sites were identified endoscopically and treated with TAE at West China Hospital of Sichuan University from January 1st, 2005 to July 1st, 2021. This study was approved by the Ethics Committee on Biomedical Research of West China Hospital of Sichuan University.

### Patients

The inclusion criteria for the study population were patients who had been diagnosed with ANVUGIB by clinical features and a bleeding site found at endoscopy, and subsequently treated with TAE. Exclusion criteria were patients who had biliary or pancreatic duct hemorrhage, hemodynamically unstable patients who were dependent on intensive care support or with multi-organ failure, and digestive tract bleeding due to malignant tumors. According to the presence or absence of preplaced metal clips, they were divided into two groups.

### TAE Technical approach

The procedure was performed using a transfemoral cannulation route in which a 5–6 Fr arterial sheath was placed over the common femoral artery, followed by access to the abdominal trunk using various smaller caliber selective catheters to the common hepatic artery and superior mesenteric artery, respectively, as appropriate, for arteriography to delineate the anatomy and to determine contrast extravasation. If no contrast spillage was found, angiography was performed using a microcatheter cannulated into the gastroduodenal artery, left gastric artery, or splenic artery, depending on the information provided by the endoscopy about the possible location of the bleeding source. If contrast spillage was detected, embolization was performed using coils, N-butyl cyanoacrylate, gelfoam sponges, and embolic granules depending on the active bleeding vessel; if contrast spillage was still not detected and a metal clip was available, the blood supply vessel to the site was embolized according to its location, or if no metal clip was available, embolization was performed according to the endoscopic indication of the bleeding site.

### Variables

The outcome measurements included the clinical success rate, mortality rate, rebleeding rate, need for surgery, and hemoglobin level at discharge (g/L).

Technical success was defined as repeat of the responsible angiogram that did not show the responsible artery, and no collateral blood supply was formed immediately after embolization [6].

Clinical success was defined as control of bleeding with no need for addition endoscopic treatment, repeated embolization, or surgery for 30 days after embolization. Mortality was recorded within 30 days after TAE.

The baseline information included gender, age, history of surgery or trauma within 30 days, the use of antithrombotics, the Rockall score, the hemorrhage site, intervals from hemorrhage to TAE, angiography result, blood transfusion before TAE, hemoglobin before TAE, coagulation disorders (PLT ≤ 80 × 10^9^/L or international normalized ratio, INR ≥ 1.5, or activated partial thromboplastin time, APTT ≥ 45 s [7]) and embolization materials.

The Rockall Score is a risk stratification tool for rebleeding and mortality in upper gastrointestinal bleeding patients. The scoring criteria include the following factors: age, hemodynamic status, comorbidities, endoscopic diagnosis, and endoscopic signs of bleeding. The maximum score is 11 points, with higher scores indicating an increased risk of rebleeding and mortality.

### Statistical analysis

Continuous variables subjected to a normal distribution are reported as the mean, standard deviation and range, and continuous variables that were not subjected to a normal distribution are reported as the median, interquartile range and range. Categorical variables were reported as frequencies and composition ratios.

The comparison of two sets of sample means used the t test when continuous and subjected to a normal distribution, while the Mann–Whitney U test was used for nonnormally distributed data. The Pearson chi-square test or Fisher's exact probability was applied when the two sets of composition ratios were compared. To avoid omitting some important clinical factors, variables with *p* < 0.100 were entered into the logistic regression model and performed with “Forward: LR” method. Odds ratios (ORs), together with 95% confidence intervals (CIs), were reported to estimate the strength of association. The significance level was set at *P* ≤ 0.05, and the two-sided P values were reported. Statistical analysis was performed using IBM SPSS Statistics, version 25.0 (IBM Corp, Armonk, New York, USA).

## Result

**Fig. 2:**
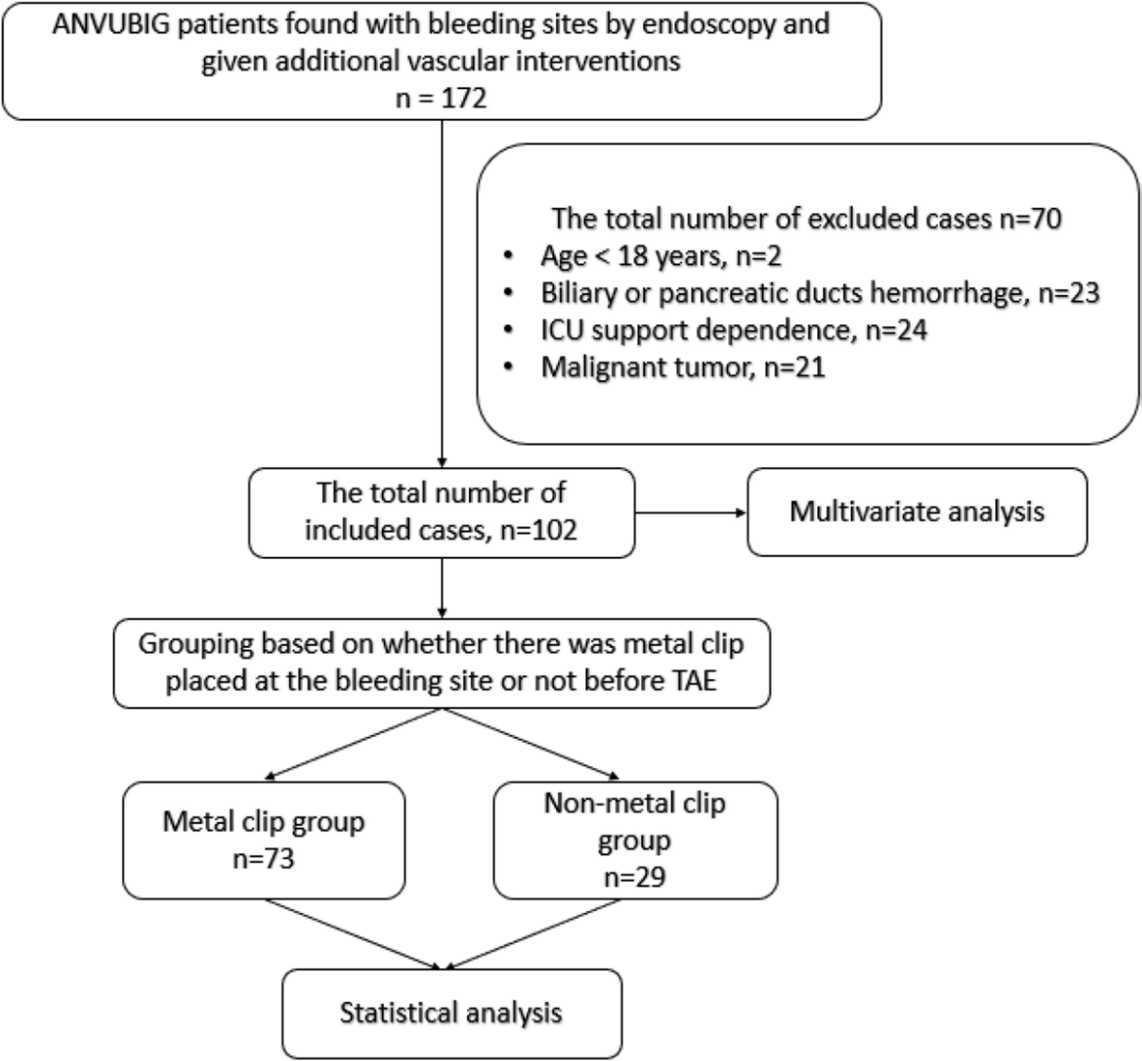
Flow diagram of study subjects

According to the inclusion criteria, 172 patients were initially screened out, and 70 patients were excluded. 102 patients were included in the study. There were 73 cases in the metal clip group and 29 cases in the no metal clip group. (Fig. 2).

There were no significant differences between the two groups in sex, age, history of surgery/trauma, history of anti-thrombotic medication, Rockall score, bleeding site, time from bleeding to TAE, angiography result, preoperative blood transfusion volume, preoperative hemoglobin, accompanying coagulation dysfunction or use of embolic materials (Table 2).

**Table 2:** Comparison of baseline data

	Metal clip group (*n* = 73)	Non-clip group (*n* = 29)	*P* value
Gender(male/female)	63(86.3)/10(13.7)	22(75.9)/7(24.1)	0.255^a^
Age(years)	61(21)[19–83]	59(46)[33–86]	0.450^d^
History of surgery/trauma(yes/no)	19(26.0)/54(74.0)	3(10.3)/26(89.7)	0.141^c^
History of anti-thrombotic medication(yes/no)	15(20.5)/58(79.5)	6(20.7)/23(79.3)	1.000^c^
Rockall score	5(3)[1–9]	5(3)[1–8]	0.337^d^
Bleeding site			1.000^b^
Cardia	3(4.1)	1(3.4)	\
Stomach	29(39.7)	12(41.4)	\
Duodenum	32(43.8)	12(41.4)	\
Multiple-site bleeding	4(5.5)	2(6.9)	\
No active bleeding	5(6.8)	2(6.9)	\
Intervals between bleeding to TAE(day)	6(10.5)[0–46]	12(21)[0–50]	0.081^d^
Angiography result(negative/positive)	55(75.3)/18(24.7)	20(69.0)/9(31.0)	0.510^a^
Preoperative blood transfusion(U)	4(8)[0–75.5]	3(6.25)[0–35.5]	0.307^d^
Preoperative hemoglobin(g/L)	^f^65.5(16.7)[16–98]	^f^68.6(22.2)[29–114]	0.473^e^
Preoperative WBC(10^9)	^f^8.8(3.8)[2.4–19.1]	^f^10.4(5.7)[4.17–24.73]	0.275^e^
Preoperative albumin(g/L)	^f^29.1(5.8)[16.0–42.7]	^f^28.0(8.5)[13–43.5]	0.620^e^
Coagulopathy(yes/no)	30(41.1)/43(58.9)	10(34.5)/19(65.5)	0.539^a^
Embolic agents			0.589^b^
Coils	38(52.1)	20(69.0)	\
N-butyl cyanoacrylate	7(9.5)	1(3.4)	\
PVA granules	1(1.4)	0	\
Coil and PVA granules	3(4.1)	2(6.9)	\
Coils and N-butyl cyanoacrylate	23(31.5)	6(20.7)	\
Coils and gelfoam sponge	1(1.4)	0	\
Forrest Classification			0.226^b^
Ia	11(15.1)	2(6.9)	\
Ib	33(45.2)	10(34.5)	\
IIa	12(16.4)	7(24.1)	\
IIb	14(19.2)	6(20.7)	\
IIc	2(2.7)	4(13.8)	\
III	1(1.4)	0	\
Ulcers’ long diameter(cm)			0.331^b^
0 ~ 1	29(39.7)	8(27.6)	\
1 ~ 2	27(37.0)	10(34.5)	\
> 2	17(23.3)	11(37.9)	\

Enumeration data: Frequency (percentage); Measurement data: Median (quartile range) [range]

^a^Pearson chi-square test

^b^Fisher's exact probability

^c^Continuous calibration chi-square test

^d^Mann–Whitney U test

^e^Independent sample T test

^f^Mean (standard deviation) [range]

The technical success rate was 100%, the clinical success rate was 74.5%, the mortality rate was 14.7%, the rebleeding rate was 13.7%, and the additional surgery rate was 13.7%. No TAE-related complications occurred.

The clinical success rate of the metal clip group was higher than that of the no metal clip group (82.2% vs 55.2%, respectively, *P* < 0.001), and the rebleeding rate (8.2% vs 27.6%, respectively, *P* = 0.039) of the metal clip group was lower than that of the no metal clip group. The additional surgery rate of the metal clip group was lower than that of the no metal clip group (11.0% vs 20.7%, respectively, *P* < 0.001). There were statistical significance for all of the differences. The mortality rate in the metal clip group was reduced compared with that in the no metal clip group (11.0% vs 24.1%, respectively, *P* = 0.146), although there was no significant difference, which might be related to the limited sample size. There was no statistically significant difference in hemoglobin levels at discharge, postoperative minimum hemoglobin, postoperative blood transfusion, postoperative organ failure, postoperative hospital stay and total expense. (Table 3).

**Table 3:** Comparison of outcome measurements

	Metal clip group (*n* = 73)	Non-clip group (*n* = 29)	*P* values
**Clinical success(yes/no)**	**60(82.2)/13(17.8)**	**16(55.2)/13(45.8)**	** < 0.001**^**a**^
Death(yes/no)	8(11.0)/65(89.0)	7(24.1)/22(75.9)	0.146^a^
**Rebleeding(yes/no)**	**6(8.2)/67(91.8)**	**8(27.6)/21(72.4)**	**0.039**^**a**^
**Additional surgery(yes/no)**	**8(11.0)/65(89.0)**	**6(20.7)/23(79.3)**	** < 0.001**^**a**^
Discharged hemoglobin(g/L)	^f^84.3(16.2)[27–125]	^f^85.4(27.1)[43–138]	0.360^e^
Postoperative minimum hemoglobin(g/L)	^f^65.0(17.3)[24–122]	^f^60.7(15.0)[36–94]	0.251^e^
Postoperative blood transfusion(U)	2(5)[0–28]	4(6.8)[0–38.5]	0.140^d^
Postoperative organ failure(yes/no)	10(13.7)/63(86.3)	3(12.7)/26(87.3)	0.897^a^
Postoperative hospital stay(Day)	10(10)[2–124]	12(10)[0–41]	0.696^d^
Hospitalization total expense(CNY)	53,810.8(72,206.7)[10411.0–839485.34]	54,385.0(63,355.1)[24039.5–243,034.0]	0.762^d^

Enumeration data: Frequency (percentage); Measurement data: Median (quartile range) [range]

^a^Pearson chi-square test

^b^Fisher's exact probability

^c^Continuous calibration chi-square test

^d^Mann–Whitney U test

^e^Independent sample T test

^f^Mean (standard deviation) [range]

### Analysis of the factors influencing clinical success

A univariate analysis of the factors influencing clinical success showed differences in Rockall score (5 vs 7, respectively, *P* = 0.016) and metal clip placement rate (78.9% vs 50%, respectively, *P* = 0.005) between the clinical success group and clinical failure group. Multivariate analysis showed that metal clip placement at the bleeding site was a protective factor for clinical success. The probability of clinical success for TAE hemostasis in patients with metal clips placed at the bleeding sites was 3.75 times higher than in those without metal clip placement (OR = 3.750, 95CI = 1.456–9.659, *P* = 0.004). (Table 4).

**Table 4:** Comparison of risk factors

	Clinical success *N* = 76	Clinical failure *N* = 26	*P* value	OR(95CI)	*P*’ value
gender(male/female)	64(84.2)/12(15.8)	21(80.8)/5(19.2)	0.762^a^	\	\
age(years)	60(23.25)[19–83]	58(33.25)[22–80]	0.776^d^	\	\
History of surgery/trauma(yes/no)	15(19.7)/61(80.3)	7(26.9)/19(73.1)	0.442^a^	\	\
History of anti-thrombotic medication(yes/no)	15(19.7)/61(80.3)	6(23.1)/20(76.9)	0.934^c^	\	\
Angiography(negative/positive)	54(71.1)/22(28.9)	21(80.8)/5(19.2)	0.332^a^	\	\
**Metal clip (yes/no)**	**60(78.9)/16(21.1)**	**13(50)/13(50)**	**0.005**^**a**^	**3.750(1.456–9.659)**	**0.004**
**Rockall score**	**5(2)[1–8]**	**7(3.25)[2–9]**	**0.016**^**d**^	1.407(1.066–1.856)	0.111
Intervals between bleeding to TAE(day)	6(13.75)[0–46]	8(10.25)[0–50]	0.416^d^	\	\
Preoperative blood transfusion(U)	4(7.5)[0–75.5]	4(11.5)[0–35.5]	0.601^d^	\	\
coagulopathy(yes/no)	30(39.5)/46(60.5)	10(38.5)/16(61.5)	1.000^a^	/	

Enumeration data: Frequency (percentage); Measurement data: Median (quartile range) [range]

^a^Pearson chi-square test

^b^Fisher's exact probability

^c^Continuous calibration chi-square test

^d^Mann–Whitney U test

^e^Independent sample T test

*P*’ value: Logistic regression (Forward: LR)

^f^Mean (standard deviation) [range]

## Discussion

The clinical treatment of ANVUGIB may be challenging. Endoscopic examination is always required for these patients with hemostasis implemented if necessary and possible. However, there is still a high rebleeding rate after endoscopic treatment [2]and patients who have failed endoscopic hemostasis are likely to be treated byTAE. However, it is often difficult to locate the responsible blood vessel during TAE. Previous case reports have shown that preplaced metal clips under endoscopy can play a role in locating the responsible blood vessels during TAE [5, 2, 6]. However, these studies were all case series. This study is the first study to assess the impact of endoscopically-placed metal clips at the bleeding site of ANVUGIB patients on the clinical effect of TAE. In previous related studies, advanced age [8], history of trauma or invasive surgery [9], use of antithrombotic drugs [10], bleeding site [11], ulcer’s diameter [11], longer time to TAE [7, 12], red blood cell suspension ≥ 6 U infusion before TAE [13–15], low hemoglobin [13], coagulation dysfunction [14–16], different embolic materials [13] and angiography result [17] were reported to affect prognosis of ANVUGIB patients after TAE treatment. In this study, these factors were comparable between the metal clip group and the no metal clip group.

Compared with the no clips group, the group with metal clips had a higher clinical success rate. The metal clip group also had a lower rebleeding rate and additional surgery rate. Consistent with these results, metal clip marking was also shown to improve overall clinical success, patients marked with metal clips were 3.75 times more likely to achieve successful hemostasis by TAE. These results indicated that a metal clip placed at the bleeding site was a facilitating factor for the success of hemostasis by TAE by locating bleeding vessels and guiding embolization during TAE procedures. Due to the diversity of vascular anatomy, empirical vascular embolization often fails to cover the responsible vessel and results in failure of hemostasis. In angiographically negative patients, metal clip marking was demonstrated to increase the accuracy of angioembolization by indicating the location of the bleeding lesion and suggesting the responsible vessel [7]. In addition, the metal clip probably improves the rate of positive angiography by shortening the distance between the location of the contrast release and the bleeding site in selective angiography [2]. Moreover, metal clip marking could shorten the angiographic operation time [6]. Therefore, it is recommended that metal clip labeling should be performed if there is a possible TAE.

A meta-analysis [7] including 819 patients who underwent TAE without metal clip marking reported an overall mortality rate of 28%. This was higher than the mortality rate of both the marking and nonmarking groups in this study. There were many factors that might be related to the mortality rate, such as coagulation dysfunction and interval between bleeding and TAE. In our study, the lower mortality rate might be attributed to all patients included in this study undergoing endoscopy prior to TAE, and the site of bleeding was detected endoscopically, which was helpful to guide angiography and embolization. In addition, patients who did not undergo endoscopy due to unstable vital signs of bleeding and went straight to emergency TAE, as well as patients whose bleeding site was not observed endoscopically due to massive blood retention in the gastrointestinal tract, were excluded, as these patients were often bleeding more heavily and in a more critical condition. The mortality rate in the metal clip group was less than half that in the nonclip group, but this result was not significantly different. The insignificant difference may be related to the limited sample size, which was due to the exclusion of a large number of patients who had undergone angiography only, without arterial embolization.

Many studies have discussed factors related to recurrent bleeding. Lau et al. [18] defined the criteria for high risk of recurrent bleeding as ulcers ≥ 20 mm, spurting bleeding, hypotensive shock or hemoglobin < 90 g/L. Patients who fulfilled one or more of the above criteria had a risk of at least 16.7% to rebleed. Kaminskis et al. [19] used Forrest type Ia-IIb and Rockall score ≥ 5 as standards to define whether patients were highly likely to rebleed. These criteria are used to determine the need for TAE and to assess the need for endoscopic hemostasis. Previous studies also showed that the failure of endoscopic hemostasis was related to the exposure of arteries, ulcers and adhesive blood clots, massive bleeding or large ulcers combined with active bleeding [2].

In this study, no TAE-related complications occurred in either the metal clip group or the no metal clip group. Arterial embolization in the GI tract above the duodenal suspensory ligament is generally considered to be very safe, as the stomach and duodenum have abundant collateral circulation; thus, serious complications such as gastrointestinal ischemia are rare [7]. In our study, no extra bleeding or other complications occurred due to the placement of the metal clip. The guidelines for the diagnosis and treatment of ANVUGIB [20] recommend that patients with ANVUGIB should accept endoscopy or treatment within 24 h after bleeding. The metal clip can act both as a hemostasis tool and embolization label, and the marking can be performed at the same time as the metal clip used for endoscopic hemostasis. For patients who underwent endoscopic hemostasis by electrocoagulation or other methods, a metal clip could also be placed at the edge of the lesion. It does not require additional examination. Therefore, the use of metal clips for marking is safe and convenient.

**Fig. 3:**
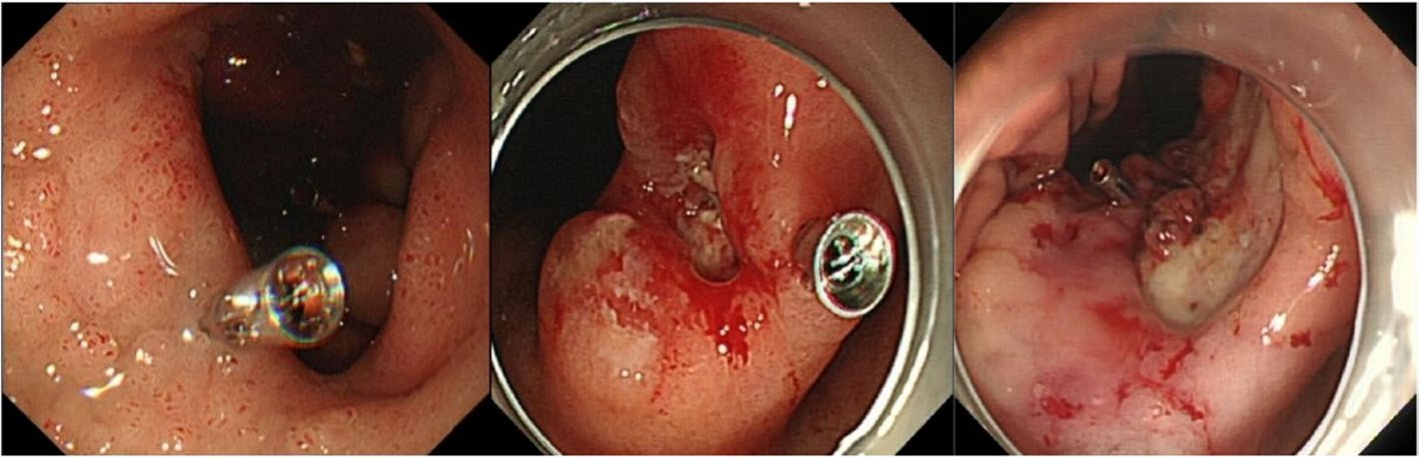
Images of endoscopic metal clip markings

Although no consensus has been reached, the overall view of these studies is basically the same. Combined with the results of our study, we recommend that lesions with a Forrest classification of Ia-IIb and a Rockall score ≥ 5 should be markedwith metal clips when conditions permit. Metal clip marking could be performed either by direct metal clip hemostasis or by marking at the edge of the lesion without hemostasis. (Fig. 3).

Limitations of this study are the inherent flaws of retrospective research, the operators and other factors that may affect the results cannot be controlled comprehensively; the data that can be collected are limited, such as the operation time and other important outcome indicators cannot be collected; and the bias of a single-center study is difficult to avoid.

## Data Availability

All data analyzed during this study are included in this published article.
